# The pattern of orbital walls fractures in north of Jordan

**DOI:** 10.4317/jced.60894

**Published:** 2023-10-01

**Authors:** Anwar B. Bataineh

**Affiliations:** 1 BDS, MScD, MDSc, CSOS. Professor of Oral & Maxillofacial Surgery, Faculty of Dentistry, Jordan University of Science & Technology

## Abstract

**Background:**

The aim of this study was to examine the incidence and demographic characteristics of patients with orbital walls fractures who were treated in the department of oral and maxillofacial surgery of a tertiary hospital in Jordan.

**Material and Methods:**

A retrospective cohort study of patients with a diagnosis of with selected orbital walls fracture was designed and implemented, during a two-year period between January 2020 and December 2021. Information on patients treated for orbital walls fractures were retrieved and analyzed regarding age, sex, etiology, anatomical site, and treatment modality. Descriptive data presented as simple frequencies and percentages.

**Results:**

A total of 76 patients with 100 orbital wall fractures, age range was 4-68 years old with a mean age 28 (SD±12), of whom were 53 (69.7%) were male and 23(30.3%) were female, with male:female ratio was 2:1. The most common etiology of all orbital walls fractures was RTA in 47 patients (61.8%; *P* ≤0.021), followed by violence in male patients. The most prevalent age-group was 21-30 years old with 16 patients (21.05%; *P* ≤0.235), and single orbital wall fractures 56 (56%) were more common than combined orbital wall fractures.

**Conclusions:**

In conclusion, this study will contribute to identifying the best clinical therapy and protective techniques for individuals with orbital fractures.

** Key words:**Orbital fracture, Etiology, Orbital trauma, Road traffic accidents, Trauma.

## Introduction

Orbital fracture usually occurs frequently because of blunt orbital and midfacial traumas and may involve ocular injuries. In general, patients are polytraumatized and their functional and cosmetic treatments are performed in different medical specialties such as ophthalmology, otorhinolaryngology, neurosurgery, plastic and reconstructive surgery and oral and maxillofacial surgery. Priority that these injuries is the wellbeing of the world since orbital trauma is the second most common cause of blindness.

The degree of a fracture can ranges from minor, barely displaced fractures of a single wall that don’t need surgery to significant orbital displacement. There are many different types of orbital fractures that can occur, but they can be broadly divided into two categories: pure and impure. Blowout fractures are another name for pure orbital fractures. Blowout fractures are breaks in the internal orbital walls or floor that do not also involve a break in the rims of the orbit. The floor and/or medial walls of the orbit, which are the thinnest parts of the wall, are where orbital blowout fractures most frequently occur.

Impure orbital fractures are those that affect the internal orbit and include zygomaticorbital, nasorbitoethmoid, Le Fort, and supraorbital rim fractures. Fractures of the zygomatic complex are the most frequent orbital fractures, and impure orbital fractures are more common than pure ones (1,2). Additionally, there is a strong correlation between these conditions and injuries to the eyes and brain (3). As a result, triage mandates that orbital fracture treatment might be postponed until other critical functions or structures have been treated (4-6).

Many epidemiological studies of orbital fracture have been described in the literature, with reports of variable diagnostic criteria, medical and surgical treatment modalities depending on whether the patient primarily consulted an ophthalmological or oral and maxillofacial surgery clinic (7-9). These variations may also be explained by differences in the socioeconomic and cultural levels of the populations studied. Moreover, there are no recent studies on the epidemiology and etiology of orbital fractures in the middle east. The aim of this study was to examine the pattern and demographic characteristics of patients with orbital walls fractures who were treated in a tertiary hospital in north of Jordan.

## Material and Methods

The study conducted at the Department of Oral and Maxillofacial Surgery at King Abdullah University Hospital/Jordan University of Science and Technology. Ethical approval was exempted by the Institutional Ethical Review Committee of Jordan University of Science and Technology due to the retrospective nature of this study and conducted according to the Declaration of Helsinki.

A retrospective cohort study of patients with a diagnosis of with selected orbital walls fracture was designed and implemented, during a two-year period between January 2020 and December 2021. Clinical and radiologic analysis were used to diagnose orbital fractures, every patient in this had plain radiographs or Computerized Tomography Scans. The discovery of bone discontinuity during radiologic analysis supported the diagnosis. The primary outcome was the type of orbital walls fractures and secondary outcomes were etiology of orbital walls fractures.

Inclusion criteria involved all diagnosed clinically and radiographically patients for whom all records were complete with orbital fractures whether admitted to the hospital or treated as outpatients. Exclusion criteria were patients for whom clinical records were incomplete, had a history of previous fracture, other forms of maxillofacial injury isolated orbital fracture.

Information on patients treated for orbital walls fractures were retrieved and analyzed regarding age, sex, etiology, anatomical site, and treatment modality. Etiology is classified into RTA, violence, fall, and sports. Anatomical sites orbital walls fractures were classified as medial wall, inferior wall, lateral wall, and superior wall. The types of orbital walls fractures were classified as single orbital wall fractures involving one orbital wall or combined orbital wall fractures involving more than one orbital wall. Treatment modality was classified as surgical reconstruction performed with plates or conservative. All patients were referred to ophthalmologists to rule out any intraocular trauma.

Data statistically analyzed using IBM SPSS Statistics software, (Version 28; IBM, NY, USA). Descriptive data presented as simple frequencies and percentages. A Chi-Square test was performed to compare proportions, and the level of significance was set at *p* ≤0.05.

## Results

A total of 76 patients with 100 orbital wall fractures, age range was 4-68 years old with a mean age 28 (SD±12), of whom were 53 (69.7%) were male and 23(30.3%) were female, with male:female ratio was 2:1.

Distribution etiology of orbital walls fracture by patient age and sex, the associations between patient age group and etiology of orbital walls fracture, the most common etiology of all orbital walls fractures was RTA in 47 patients (61.8%; *P* ≤0.021), followed by violence in male patients 12 (15.8%) as shown in (Table 1).

**Table 1:**
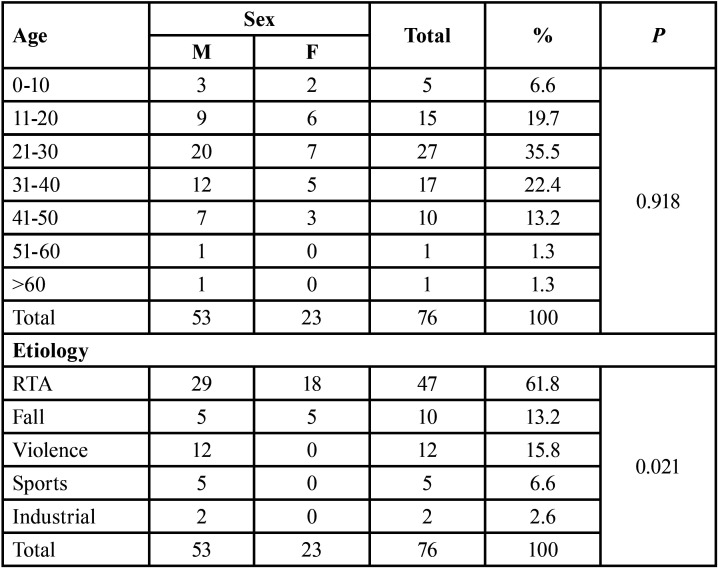
Distribution etiology of orbital walls fracture by patient age and sex.

 Table 2 summarize frequency distribution sites, type and etiology of orbital walls fracture in relationship to sex and age group, the most prevalent age-group was 21-30 years old with 16 patients (21.05%; *P* ≤0.235). The most affected site was inferior wall with 60 patients (60%; *P* ≤0.599), while the medial wall was 11 patients (11%; *P* ≤0.161). Distribution of type of orbital fracture in relationship to sex and age group, of patients with verified orbital walls fracture 69.7% were male and 30.3% were female.

**Table 2:**
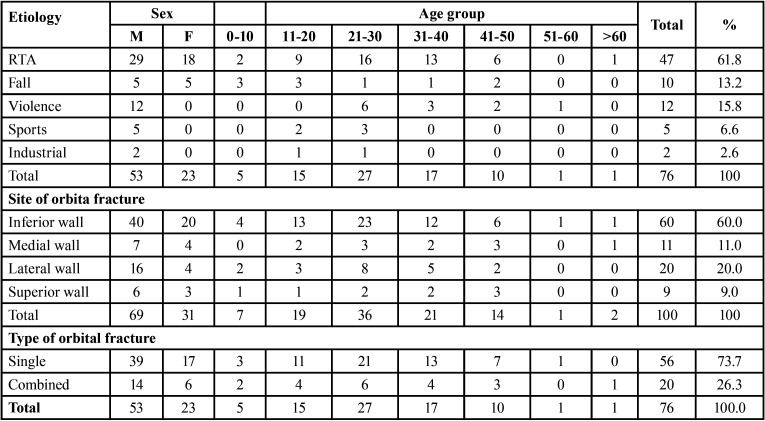
Distribution sites and etiology and type of orbital walls fracture in relationship to sex and age group.

Distribution of orbital walls fractures in Table 3, single orbital wall was commonly affected the inferior wall in 41(73.2%) cases, in combined orbital walls, was medial wall + Inferior wall in 5(26.3%) and in combination of single and combined orbital walls, inferior wall was mostly affected in 60(60%) cases.

**Table 3:**
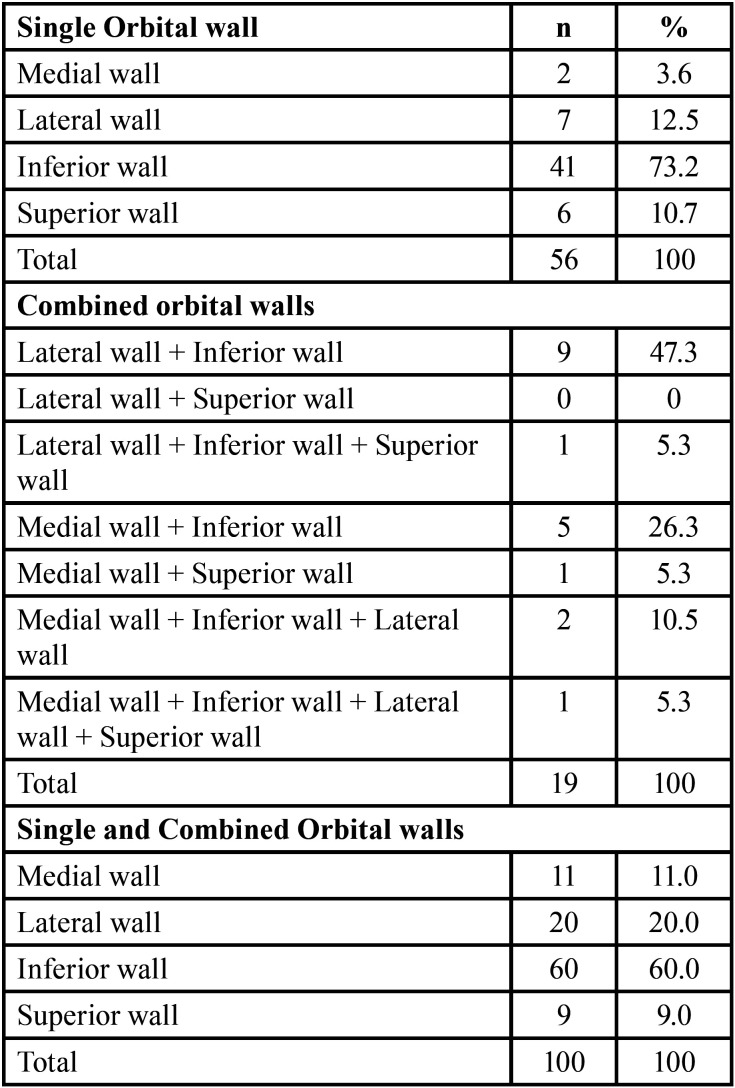
Distribution of orbital walls fractures.

Distribution of treatment modality were surgical in 47 patients (61.84%) and conservative 29 patients (38.16%) as shown in (Table 4).

**Table 4:**
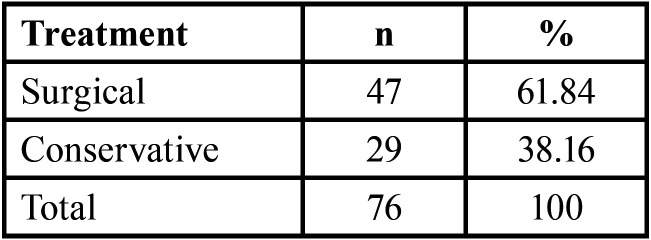
Distribution of treatment modality.

## Discussion

The etiology of orbital walls fractures differs depending on the socioeconomic and cultural backgrounds of the population being examined, as well as the nation and region where the study is being undertaken. The occurrence of orbital wall fractures is primarily influenced by two factors. Both the buckling theory and the hydraulic theory mechanisms may contribute to orbital walls fractures, according to biomechanical research carried out on cadavers (10). A trauma to the orbit accounts for roughly 3% in the United States (11). An orbital walls fractures accounts roughly for 3% in the United States (11), and approximately 16% of maxillofacial fractures were orbital walls fractures in Europe (12).

The main cause of orbital walls fractures, according to the epidemiological literature, was road traffic accidents. This was followed, in decreasing order of incidence, by violence, falls, and sports injuries (13,14). Road traffic accidents are the most frequent cause of orbital walls fractures in urban areas, according to Cruz and Eichenberger’s review (15). The most common cause of all orbital walls fractures in the current study was RTA, followed by violence only in male patients, which is consistent with the study that found the etiology of orbital walls fractures to be RTA, assaults, and falls, in decreasing order of frequency (16). Contrarily, Shere *et al*. (17) the study conducted on American soldiers found that assault was the most common etiology, followed by RTAs. The social and cultural disparities of the local community under study are what account for this discrepancy (1-3).

Blunt maxillofacial trauma frequently causes orbital walls fractures, which mostly affect males in their thirties (10,17,18). This study demonstrated that male patients were more likely than female patients to experience orbital walls fractures. These results support those of another research that has already been published (19-22). According to this study, the age range with the highest prevalence was 21 to 30 years old. Other studies have revealed that men in their thirties are most affected by orbital walls fractures, which frequently originate from blunt maxillofacial trauma (10,17,18). Assault, followed by RTA and falls, is the leading cause of orbital trauma in adults (10,13,17-19,23,24). Sports injuries and daily activities are more frequent in pediatric patients (14,15).

This current study confirmed previous research that the inferior wall is the most affected orbital wall fracture and that the inferior wall is also the most frequently afflicted orbital regions (19). Contrary to the literature, it was discovered that medial wall fractures were more common (16). This discrepancy might be caused by the fact that medial wall fractures are underdiagnosed because they do not have any symptoms (23).

The rate of single wall fractures was 47.1% in the 391 orbital fracture patients reported by Hwang *et al*. (24) and the prevalence of combined multiple bone fractures was 52.9%. In this study found that the rate of single orbital walls fractures was 56 fractures (56%), similarly to the study that discovered the incidence of single orbital walls fractures to be 57.6% (16). Most of these cases showed more than one orbital wall fracture, these findings concur with prior reports about patients with isolated head and orbital trauma (25,26).

It can vary between nations and hospitals in terms of the number and variety of specialists treating orbital fractures (27,28). An ophthalmologist and oral and maxillofacial surgeon who are specifically trained in examining the orbit after injury and determining the best treatment plan. Even that approach to treating orbital fractures is debaTable among oculoplastic and maxillofacial surgeons. Cases of mildly or non-displaced orbital walls fractures should only be observed.

Treatment options depending on the severity of the injury, when there was a sTable fracture, no enophthalmos, no muscle-orbital soft tissue compression, and when surgical intervention was declined by the patients, a conservative strategy was taken. In the present study, 29 patients (38.16%) received conservative medical care in accordance with the treatment protocol recommended for applying cold therapy, elevating the patient’s head, administering systemic and local antibiotherapy, and anti-inflammatory medication. There were 47 patients (61.84%) who were treated by surgical intervention with orbital walls fractures. According to the literature, it is observed that 80% to 86% of combined orbital wall fractures were treated by surgical intervention, Burm *et al*. (29) reported that 80.9% of combined wall fractures and Eun *et al*. (30) reported that 82% of combined wall fractures.

## Conclusions

The etiology of orbital fractures differs depending on the socioeconomic and cultural backgrounds of the population being examined, as well as the nation and region where the study is being undertaken. In conclusion, this study will contribute to identifying the best clinical therapy and protective techniques for individuals with orbital fractures.
